# A Fresh Start

**Published:** 1889-10-12

**Authors:** 


### The Doctor's Pulpit.

### A FRESH START.

When a man is recovering from a severe illness, like typhoid fever or inflammation of the lungs, if his constitution be naturally good, and the structural changes produced by the illness have not been of too extensive or profound a character, his appetite, his spirits, and his strength will progress from day to day as if by leaps and bounds. The vital energies have been for a time "chained up," so to speak; and during the period of their imprisonment have accumulated like a flowing river behind an obstructing dam. When the dam is removed by the cessation of the acute symptoms, the imprisoned energies are again let loose, and flood the whole being with their resistless flow. Life and health have made a "fresh start." There are certain senses in which it is true that man is a being peculiarly designed for fresh starts. It is also equally true that fresh starts are more than disagreeable to some men; they are hateful and practically impossible. But the man who has too great a repugnance to fresh starts may regard himself, morally and intellectually at any rate, as a kind of extinct dodo. The power to make fresh starts, the willingness to set about them, and the cheerfulness with which they are taken in hand, is an excellent measure of the amount of youthfulness that still remains to a man. A good many men—alas, for them—are quite old at fifty; a few of Fortune's special favourites, or, should we not rather say, of happy disposition and wholesome life? are quite young at seventy. All sane and healthy men are young at seventy in the sense that they are mentally prepared to reconsider any subject in heaven and earth, and if they find they have been seventy years in error, they are willing to make a fresh start.

Hardly anything is more conducive to healthy life than intelligent order; and, on the other hand, nothing is more favourable to mental and physical decay than unchanging and unthinking routine. Worry is dangerous, but stagnancy is fatal. Few things better illustrate this than reverses of fortune. A man has been bred to his father's business, has succeeded him in it, and has gone on until middle life, prosperous, contented, commonplace, and insignificant. A stroke of adverse luck has deprived him of all his possessions and left him bare as a field of hop-poles after all the pickers have gone home. A brief period of despair has given birth to a sturdy resolution. He is a man, and what may be possible for a man that he will do. He begins the world afresh, and in no long time proves to his contemporaries that, though prosperity insisted on showing him a graduate of the vast university of common-place, adversity has revealed him in his truer character of hero. Heroes are a little at a discount in these days of Professor Huxley and Mr. Andrew Lang. Perhaps, however, the loss is not on the side of the heroes.

Nature has an invincible passion for movement and life. Dried parchment is her abomination. Laws, social customs, religious formalities, have all tried their utmost to enswathe and imprison, or at least to fashion mankind after one and the same pattern. Even medicine, forgetting the vitalities of physiology, has too often pinned its faith to the crude generalisations of a dead empiricism, and has tried to make of man a mere feathered shuttlecock for the antagonistic battledores of "scientific experiment" and barefaced "rule of thumb." Man is the puppet, in turns, of society, of law, of physic, and of hierarchs; but he is not the slain victim of any. Nature—living, working, growing, expanding—sooner or later delivers her human Samson from them all. Mighty health is the parent of freedom, the living source and fountain of perennial pleasure. It is better than books, better than learning, better than wealth, better than all things. Many a lusty beggar as he crosses the domain of a gouty and irascible Lord Chancellor despises his flannel-toed lordship in his heart, and thanks Providence that he is a sound-livered tramp.

It is easy to understand how the Huns and the Goths swept away the older civilisations of Europe. Much as the world of culture despises the "Goth," the sympathies of the physiologist cannot but be largely with him. If it be permissible for an artist to admire pictures, and a Churchman to venerate saints, it is surely entirely natural and right that the physiological physician—let the qualification be noted—it is surely in every way natural and right that he should be filled with admiration for the strong-limbed, sound-hearted, clear-headed, and wholesome-tempered man who embodies and makes visible the noblest of all Nature's works. Let no skimmer of books call this "Paganism" or "Hellenism." Not that either "Paganism" or "Hellenism" is an epithet which any sane man will regard as necessarily injurious. But let every man consider in himself whether resistless health of body and mind, with the courage and perpetual youth that spring from it, is not one of the most supremely admirable gifts a reasonable Providence can endow us with. Is it possible to imagine a better immortality even than one of perfect strength and wholesomeness of body and of mind?

What is the moral of all this: what Simeon and the earlier Puritans used to call "the application"? It is that men should thoroughly understand the value of health, and that they should resolve to win it at all reasonable cost. For this purpose many men and women, nearly all those who dwell in towns, will be under the absolute necessity of sometimes making a "fresh start." Health is not merely of value as making for long life, but as producing happiness and the power of doing the best work. Happiness, we conceive to be an essential factor in life, notwithstanding the well-known disclaimer of Carlyle. Life to be successful in the highest sense must be in the main happy, and that it cannot be without health. This sounds paradoxical, no doubt; but it will be found to be on the whole quite true if thoroughly considered.

The practical question is, what is the fresh start to consist of? It may be that a complete rearrangement and new plan of work will be needed. For some people it will be sufficient to eat less or to eat more wisely; others may require to eat more. For not a few, total abstinence should constitute the new start; for a very few total abstinence ought, perhaps, to be abandoned. Some undoubtedly require an improvement of morals before they can be well; others may need to be less austere. Frivolity and waste of time are the ruin of some, and for these the new start will consist in a sober purpose soberly carried out. All work and no play is the motto of others, a motto that surely means ill-health and a spoiled temper sooner or later. Play, sometimes even "The Play," is the change to which such will do well to resort.

It is not really necessary to go minutely into imaginary circumstances and cases. The average Briton, if he takes the trouble to think over his affairs, generally knows where the shoe pinches, and where the fresh start should be made if things are to run smoothly for his health. It is not so much knowledge that is required as energy of purpose. Has a man or a woman force of character sufficient for the initiation of a new start? That is really the question of questions. He can see where he has been wrong; he can see where he still is wrong; can he pull himself together and then pull himself up? The whole difficulty turns on that pivot. The religious preacher justifies all sorts of appeals to the reason and conscience by the consideration that he is arguing with a man "for his life." It is exactly so with the doctor who has any kind of reasonable enthusiasm for his "calling" and his work. All sorts of feeble health and wrong habits have an ultimate bearing on "the life." Life may be strong, ideal, happy, perfect. It may also be feeble, foolish, inconsequent, wretched, and a failure. Whether it be the one or the other depends, it may almost be said entirely, on the health. Can it be wondered at, therefore, that the physician who sits down to write should almost unconsciously assume the tone of the preacher? It is an altogether practical question for every man to decide whether his life and health be the best they might be, or whether they are quite away down below any worthy level. If they are such as to bring him neither happiness nor success, but the opposite, the least he can do, being a rational creature, is to consider in what way, and at what earliest possible moment, he can make a "fresh start."

